# Evaluation of right ventricular function and driving pressure with blood gas analysis could better select patients eligible for VV ECMO in severe ARDS

**DOI:** 10.1186/s13054-021-03646-x

**Published:** 2021-06-27

**Authors:** Matthieu Petit, Armand Mekontso-Dessap, Paul Masi, Annick Legras, Philippe Vignon, Antoine Vieillard-Baron

**Affiliations:** 1 Medical Intensive Care Unit, APHP, Ambroise Paré Hospital, Boulogne-Billancourt, France; 2 INSERM UMR 1018, Clinical Epidemiology Team, CESP, Université de Paris Saclay, Villejuif, France; 3 Medical Intensive Care Unit, APHP, Henri-Mondor Hospital, 94010 Créteil, France; 4 CARMAS, Paris Est Créteil University, 94010 Créteil, France; 5 INSERM, IMRB, Paris Est Créteil University, 94010 Créteil, France; 6 Medical-Surgical Intensive Care Unit and CIC 1435, Limoges University Hospital, 87000 Limoges, France; 7 Medical Intensive Care Unit, Tours University Hospital, Tours, France

**Keywords:** Acute Respiratory Distress Syndrome, ECMO, Acute cor pulmonale

## To the Editor,

The Acute Respiratory Distress Syndrome (ARDS) is still associated with high mortality [1], despite application of recent guidelines [2, 3]. The EOLIA study suggested that Extra- Corporeal Membrane Oxygenation (ECMO) could be effective in some of the most severe patients, but failed to demonstrate a 20% increase in survival [4]. One reason could be that criteria for selecting patients were only based on blood gas analysis. Our hypothesis is that adding other factors could allow a better selection of patients who could benefit from ECMO.

We took advantages to have a large multicentric cohort of patients under protective ventilation for moderate-to-severe ARDS [5] to determine the incidence, characteristics and outcome of patients eligible for ECMO according to EOLIA-based criteria and to identify patients who would benefit the most of the technique. ECMO was only used in these centers as a recue therapy.

Mechanical ventilation was applied in the volume-assist control mode, with a target tidal volume (TV) of 6–8 mL/kg (predicted body weight) and a plateau pressure < 30 cmH_2_O. Respiratory rate could be increased in case of high arterial carbon dioxide partial pressure (PaCO_2_). Prone positioning was left to the discretion of the attending physician, but was typically performed in patients with a PaO_2_/FiO_2_ < 100 mmHg and/or an acute core pulmonale (ACP) [6]. Patients eligible for ECMO according to EOLIA-based criteria were identified as follows: PaO_2_/FiO_2_ < 80 mmHg with optimal PEEP, or a pH < 7.25 and PaCO_2_ > 60 mmHg with a respiratory rate ≥ 35 cycles/min, despite the use of prone positioning or nitric oxide inhalation.

Statistical analysis was performed with R.4.0.4. Patients eligible for ECMO were compared to the rest of the cohort. Continuous data, expressed as medians (interquartile ranges), were compared with Mann–Whitney test. Categorical variables, expressed as numbers and percentages, were compared using the chi-square test or Fisher exact test. To evaluate independent factors associated with ICU mortality in this identified subgroup of patients, significant or marginally significant (*p* < 0.10) bivariate risk factors were examined using univariate and multivariable backward stepwise mixed logistic regression stratified on the center. SAPS II was forced in the model.

752 patients were studied. Characteristics and outcome are given in the Table 1. 67 (9%) patients were potentially eligible for ECMO. They had lower PaO_2_/FiO_2_ (62 [55–72] versus 114 [90–120] mmHg: *p* < 0.01) and higher incidence of ACP (42% versus 20%, *p* < 0.001). Only 8 of them underwent the procedure. In-ICU mortality in the whole cohort was 36%. Causes of death in patients eligible for ECMO was multi-organ failure in 21 (68%), neurologic in 4 (13%) and ECMO complication in 3 (10%). Only 3 patients (10%) died from hypoxic cardiac arrest.

**Table 1:** Clinical characteristics and outcome of the entire cohort according to EOLIA criteria

Characteristics, outcomes and complications	ARDS patients without EOLIA criteria (*n* = 685)	ARDS patients with EOLIA criteria (*n* = 67)	*p*-value
Age (years)	59 (47–72)	56 (47–70)	0.43
Male sex, *n* (%)	465 (68)	42 (63)	0.47
SAPS II	51 (38–65)	47 (33–64)	0.27
Weight (kg)	77 (65–84)	70 (59–86)	0.43
Cause of ARDS, *n* (%)			0.20
Pneumonia	83 (12)	8 (12)	
Aspiration	259 (39)	34 (51))	
Non-pulmonary sepsis	245 (37)	17 (26)	
Other causes	83 (12)	7 (11)	
Respiratory setting at inclusion			
Tidal volume (ml/kg)	6.7 (6.0–8.0)	6.02 (5.4–6.9)	< 0.01
Respiratory rate (cycle/min)	22 (16–27)	26 (22–30)	< 0.01
PEEP (cmH_2_O)	8 (5–10)	10 (7–12)	< 0.01
Plateau pressure (cmH_2_O)	24 (21–28)	27 (25–29)	< 0.01
Compliance (ml/cmH_2_O)	30.7 (24–39.1)	25.9 (19.3–33.8)	< 0.01
Driving pressure (cmH_2_O)	15 (13–19)	17 (14–19.8)	0.04
Arterial blood gases			
PaO_2_/FiO_2_ ratio (mmHg)	114 (90–120)	62 (55–72)	< 0.01
PaCO_2_ (mmHg)	44 (38–52)	48 (41–60)	< 0.01
Shock, *n* (%)	449 (66)	53 (79)	0.04
Prone positioning, *n* (%)	163 (24)	55 (82)	< 0.01
VV ECMO in rescue during ARDS course, *n* (%)	0 (0)	8 (12)	< 0.01
RRT during ARDS course, *n* (%)	126 (30)	15 (40)	0.27
Echocardiographic findings			
RVEDA/LVEDA	0.68 (0.57–0.81)	0.83 (0.64–1.04)	< 0.01
Systolic pulmonary artery pressure (mmHg)	35 (20–48)	52 (35–59)	< 0.01
Severe acute cor pulmonale	43 (6)	11 (16)	< 0.01
Outcome, *n* (%)			
ICU mortality	243 (36)	31 (46)	0.10
ICU stay (days)	16 (8–30)	15 (6–31)	0.43

Values are expressed as median (interquartile range) or *n* (%)

ARDS, Acute Respiratory Distress Syndrome; PEEP, Positive End-Expiratory Pressure; VV ECMO, Veno-Venous Extracorporeal Membrane Oxygenation; RRT, Renal Remplacement Therapy; RVEDA, Right Ventricular End-Diastolic Area; LVDEA, Left Ventricular End-Diastolic Area; ICU, Intensive Care Unit

Characteristics and outcome of patients potentially eligible for ECMO according to ICU mortality are given in the Table 2. In multivariable analysis, severe right ventricular dilatation (right-to-left ventricle end-diastolic area ratio > 1) and driving pressure were the only factors associated with in-ICU mortality (OR [95% CI]: 5.62 [1.44–27.39], *p* = 0.02 and 1.14 [1.01–1.31], *p* = 0.04, respectively).

**Table 2:** Clinical characteristics and echocardiographic findings of ARDS patients eligible for ECMO

Characteristics, outcomes and complications	Survivors (*n* = 36)	Non survivors (*n* = 31)	*p*-value
Age (years)	56 (45–70)	57 (48–70)	0.44
Male sex, *n* (%)	23 (64)	19 (61)	0.47
SAPS II	44 (32–58)	53 (36–75)	0.15
Weight (kg)	74 (63–97)	67 (56–80)	0.22
Cause of ARDS, *n* (%)			0.63
Pneumonia	6 (17)	2 (7)	
Aspiration	18 (50))	16 (53)	
Non-pulmonary sepsis	8 (22)	9 (30)	
Other causes	4 (11)	3 (10)	
Respiratory setting at inclusion			
Tidal volume (ml/kg)	6.3 (5.5–7.6)	5.9 (5.3–6.6)	0.13
Respiratory rate (cycle/min)	25 (20–27)	30 (25–30)	< 0.01
PEEP (cmH_2_O)	10 (8–12)	10 (7–12)	0.72
Plateau pressure (cmH_2_O)	26 (24–29)	28 (25–38)	0.07
Compliance (ml/cmH_2_O)	30 (2338)	23 (16–28)	< 0.01
Driving pressure (cmH_2_O)	16 (14–19)	19 (16–22)	0.05
Arterial blood gases			
PaO_2_/FiO_2_ ratio (mmHg)	69 (58–74)	60 (55–67)	0.08
PaCO_2_ (mmHg)	48 (40–52)	51 (42–70)	0.17
Shock, *n* (%)	25 (70)	28 (90)	0.07
Prone positioning, *n* (%)	31 (86)	24 (77)	0.52
VV ECMO in rescue during ARDS course, *n* (%)	2 (1)	6 (19)	0.13
RRT during ARDS course, *n* (%)	9 (33)	6 (55)	0.28
Echocardiographic findings			
RVEDA/LVEDA	0.71 (0.57–0.93)	0.98 (0.71–1.10)	0.10
Pulmonary hypertension (mmHg)	51 (44–55)	52 (34–63)	0.85
Severe acute cor pulmonale	3 (8)	8 (26)	0.09
ICU stay (days)	19 (14–34)	12 (3–20)	0.16

Values are expressed as median (interquartile range) or *n* (%)

ARDS, Acute Respiratory Distress Syndrome; PEEP, Positive End-Expiratory Pressure; VV ECMO, Veno-Venous Extracorporeal Membrane Oxygenation; RRT, Renal Remplacement Therapy; RVEDA, Right Ventricular End-Diastolic Area; LVDEA, Left Ventricular End-Diastolic Area; ICU, Intensive Care Unit

A limitation of our study is that eight patients of the cohort received ECMO as a rescue therapy, which may have influenced our results, especially since the technique is now safer when performed in expert centers. However, six of these eight patients died.

In conclusion, we report a 9% incidence of patients who reach the EOLIA-based criteria for ECMO in a large non-selected cohort of ARDS patients ventilated with moderate-to-severe ARDS. These patients exhibited higher driving pressure and more frequent right ventricle failure, both being independently associated with ICU mortality. How this subgroup of patients could be considered as the ideal target for ECMO selection strategy should better be evaluated in the future.

## Data Availability

The datasets used and/or analysed during the current study are available from the corresponding author on reasonable request.

## Abbreviations

ARDS: Acute Respiratory Distress Syndrome
ECMO: Extra-Corporeal Membrane Oxygenation
TV: Tidal Volume
ACP: Acute Core Pulmonale
PEEP: Positive End Expiratory Pressure
SAPS II: Simplified Acute Physiology Score II
ICU: Intensive Care Unit
RRT: Renal Replacement Therapy
RVEDA: Right Ventricular End-Diastolic Area
LVDEA: Left Ventricular End-Diastolic Area

## References

[1] Bellani G, Laffey JG, Pham T, Fan E, Brochard L, Esteban A (2016). Epidemiology, patterns of care, and mortality for patients with acute respiratory distress syndrome in intensive care units in 50 countries. JAMA.

[2] Fan E, Del Sorbo L, Goligher EC, Hodgson CL, Munshi L, Walkey AJ (2017). An official American Thoracic Society/European Society of Intensive Care Medicine/Society of Critical Care Medicine clinical practice guideline: mechanical ventilation in adult patients with acute respiratory distress syndrome. Am J Respir Crit Care Med.

[3] Papazian L, Aubron C, Brochard L, Chiche J-D, Combes A, Dreyfuss D (2019). Formal guidelines: management of acute respiratory distress syndrome. Ann Intensive Care.

[4] Combes A, Hajage D, Capellier G, Demoule A, Lavoué S, Guervilly C (2018). Extracorporeal membrane oxygenation for severe acute respiratory distress syndrome. N Engl J Med.

[5] Mekontso Dessap A, Boissier F, Charron C, Bégot E, Repessé X, Legras A (2016). Acute cor pulmonale during protective ventilation for acute respiratory distress syndrome: prevalence, predictors, and clinical impact. Intensive Care Med.

[6] Vieillard-Baron A, Prin S, Chergui K, Dubourg O, Jardin F (2002). Echo-Doppler demonstration of acute cor pulmonale at the bedside in the medical intensive care unit. Am J Respir Crit Care Med.

